# From guardian to shepherd: The novel role of p53 in collective cell migration and epithelial repair

**DOI:** 10.1002/ctm2.855

**Published:** 2022-05-11

**Authors:** Giulia Pilia, Eugenia Piddini

**Affiliations:** ^1^ School of Cellular and Molecular Medicine University of Bristol Bristol UK

**Keywords:** collective cell migration, epithelial repair, leader cells, p53

1

The effective resolution of wounds is crucial to minimise the risk that lesions pose to the organism. Wound healing is a complex process that achieves the impressive goal of sealing the breach in the tissue and reinstating its integrity via the activation of a multifaceted biological response involving hemostasis, inflammation, cell migration, cell proliferation and tissue remodelling.

A fundamental step in tissue repair is the activation of a collective migration program that ensures rapid closure of the breach by the cells at the edge of the wound before cell proliferation can restore the initial architecture. The mechanics of collective migration in epithelial wound healing have been dissected by a number of studies, many of which make use of simplified in vitro models of cultured epithelial monolayers injured by removal of a strip of cells.^1^, ^2^ Such models make it possible to investigate the dynamics and mechanisms of cell migration in tissue repair in a tractable system, where epithelial migration is isolated from the complex injury response that takes place in vivo.

The collective migration that enables epithelial repair is often initiated by a few specialized cells at the leading edge of migrating cell sheets that become migratory and guide cell migration. For this reason, these are called ‘leader cells’. Leader cells acquire a characteristic flattened morphology and activate migratory signaling cascades (such as elevation of integrin β1 and phosphoinositide 3‐kinase and increased activity of Rac1—Ras‐related C3 botulinum toxin substrate 1), which endow them with the capacity to drive migration of their neighbours.^2^, ^3^ How only a few cells from the initially homogeneous injured epithelial population are specified into leaders remained a question in the field. In a recent study, we identified an unexpected role for the tumour suppressor p53 in leader cell specification.^4^

We found that p53 levels are elevated in leader cells and, remarkably, that induction of p53 was able to instruct leader behaviour.^4^ Downstream of p53, its target p21 (also known as cyclin‐dependent kinase inhibitor 1A, CDKN1A) is also elevated in leader cells, and both p53 and p21 are sufficient and necessary to promote the leader function.^4^ Mechanistically, we could determine that p21‐dependent CDK inhibition and, consequently, cell‐cycle delay instruct leader cell specification.^4^ In fact, an asymmetry in CDK activity levels between leaders and follower is necessary for the initiation of directed migration.^4^ Accordingly, inhibiting p53 or p21 reduces the speed of migration and epithelial repair, whereas elevating p53 and p21, via irradiation‐induced DNA damage, accelerates migration and repair of injured epithelial monolayers.^4^

The discovery of the role of p53 in leader cell specification moves the question one step further: What induces p53 elevation in epithelial injury, triggering the commitment to leader fate?

Using a fluorescent reporter of p53 activity, we showed that this is increased in cells at the edge of a scratched epithelium, compared to their undamaged neighbours.^4^ Such elevation can be impaired by inhibition of p38, which has been previously reported to activate p53 upon mechanical stress.^5^ We therefore propose that mechanical damage, upon epithelial injury, induces p53 levels and consequently determines the specification of leader cells.

We then asked: What happens to leaders when the epithelium is repaired? Tracking leader cells as the epithelial gap closed, we could observe their elimination via cell competition,^4^ as previously reported for cells, which elevate p53.^5^ p53 has therefore a dual role in epithelial repair: It accelerates collective migration, by instructing leader behaviour in damaged cells, and it elicits leader cell elimination once the epithelium is repaired, ensuring that a physiological epithelial tissue architecture is reinstated.

The novel involvement of the p53‐p21‐CDK signaling axis in epithelial healing raises the question of whether such mechanism is relevant for tissue repair in vivo. Previous studies seem to support this idea. For example, the presence of a non‐proliferative migrating leading edge has been observed in wounds in vivo,^6^, ^7^ and tight spatiotemporal regulation of the balance between migration and proliferation is crucial for effective wound healing.^6^, ^7^ Analogously, during the regeneration of epicardial tissue, collective migration is led by a population of cytokinesis‐impaired cells,^8^ which are likely to elevate p53/p21 in response to incomplete cell division. Consistent with our own observation that leader cells are removed by cell competition on wound closure, some of these studies also reported that the specialised cells acting as leaders are transient, as cells carrying the leader signature are not observed once the wound has been repaired.^7^, ^8^

The presence of cell‐cycle arrested cells at the front of migrating cell populations is not limited to the context of tissue repair. During placenta formation, cytotrophoblasts penetrate the uterine wall to form the floating villi that mediate mother‐embryo exchanges.^9^ It has been reported that, as they commit to the invasive phenotype, cytotrophoblasts accumulate chromosomal abnormalities, often acquire an aneuploid karyotype and stop proliferating,^10^ all suggestive of p53/p21 activation. Similarly, angiogenesis in the developing mouse retina occurs by extension of vascular sprouts that invade the tissue following an actively migrating non‐proliferative tip cell.^11^

Given the established role of p53 in preventing neoplastic transformation and the importance of collective migration in tumour invasion and metastasis, it is tempting to question if p53‐mediated leader specification plays a role in cancer dissemination. Most human cancers carry mutations that abolish the normal function of p53. However, the tissue surrounding the tumour expresses functional p53, susceptible to be elevated by radiation and chemotherapy. It is thus possible that asymmetric expression of p53 or CDK activity in adjoining tumour–non‐tumour cell populations could result in leader‐follower behaviour, promoting tumour cell migration. This effect would explain the observation that chemotherapy‐induced senescent cells in tumour‐adjacent tissue promote metastasis^12^ and the enhanced metastasis potential observed as a side effect of radiotherapy.^13^

The discovery of p53 function in leader cell specification, if confirmed by in vivo studies, could therefore have broad implications in physiological and pathological activation of collective migration and, potentially, inform future therapeutic approaches. Figure 1.

**FIGURE 1:**
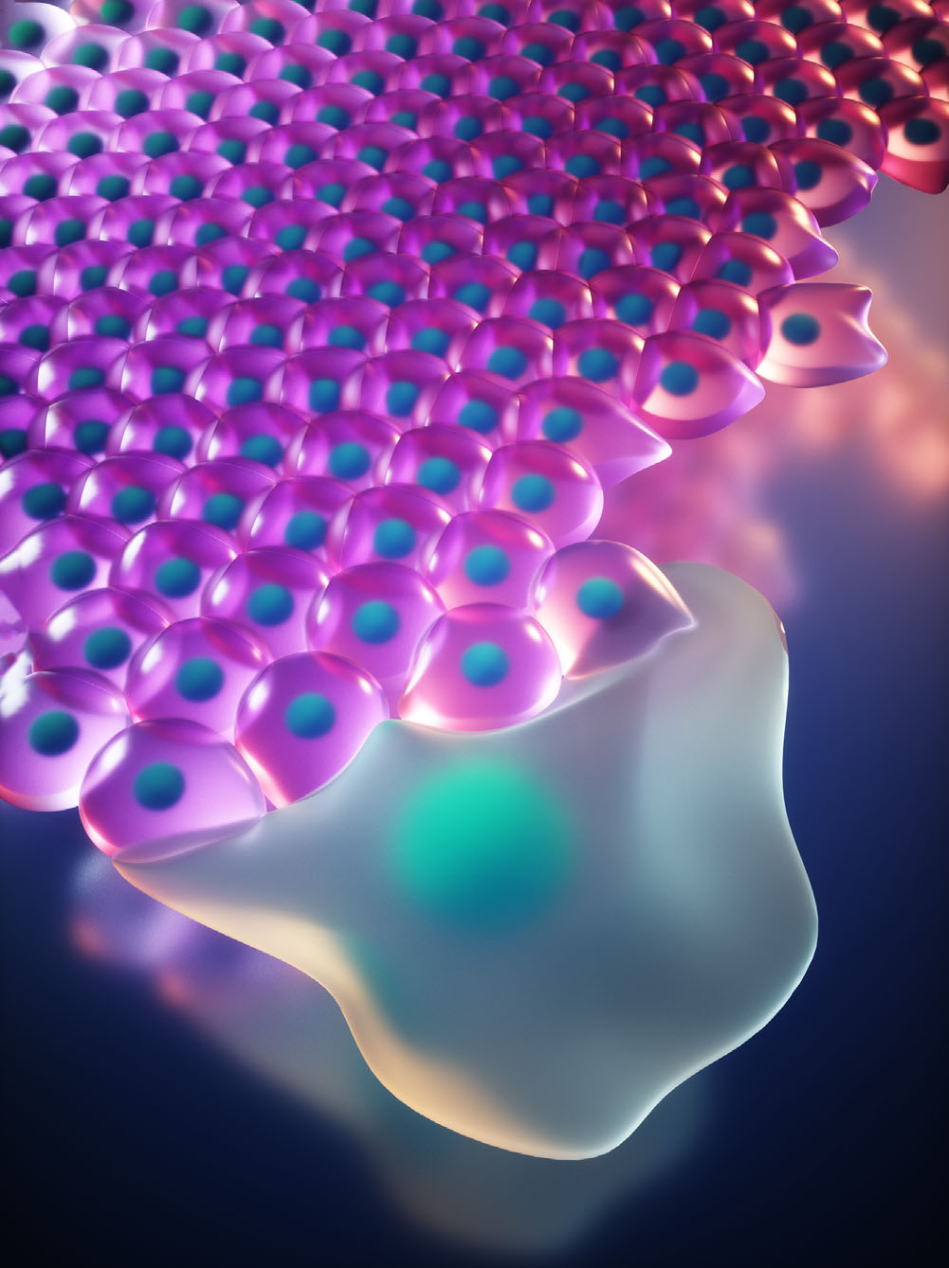
Artistic representation of a leader cell directing collective migration of its follower neighbours to facilitate epithelial repair. Credit: Giulia Pilia and Ella Maru Studio

## CONFLICT OF INTEREST

The authors declare that there is no conflict of interest that could be perceived as prejudicing the impartiality of the research reported.
